# Recombinant Human Cyclophilin A in vitro Inhibits the Formation of Fibrin Clot 

**Published:** 2012

**Authors:** Yu.A. Morozov, L.M. Khromykh, I.I. Dementieva, M.A. Charnaia, N.L. Kulikova, D.B. Kazansky

**Affiliations:** Petrovsky Russian Research Center of Surgery, Russian Academy of Medical Sciences; Carcinogenesis Institute, Blokhin Cancer Research Center; Institute of Immunological Engineering

**Keywords:** recombinant human cyclophilin A, spatial dynamics of clot growth, anticoagulant activity

## Abstract

The chemotactic properties of cyclophilin A are well-known. There exists however a poor level of understanding regarding the hemostatic effects of this protein. Herein it is shown that recombinant human cyclophilin A (rhСyA), in contrast to the granulocyte colony-stimulating factor, is capable of inhibiting*in vitro*the formation of a fibrin clot, thereby violating the spatial dynamics of clot growth; this effect is transient and dose-independent. Furthermore, the hypothesis that the conformational changes in the thrombin–rhCyA complex may mediate the anticoagulant effect of rhCyA on the autowave processes of blood clotting is postulated.

## INTRODUCTION 

Cyclophilin A is a protein with a molecular weight of approximately 20 kDa. Cyclophilin A exhibits cys-trans-isomerase activity and possesses a wide and varied range of functions. Cyclophilin A is produced by thymus cells and exhibits chemotactic activity by regulating the stem cell migration from the bone marrow to the peripheral regions [1]. Activated macrophages are also capable of secreting rhCyA, which attracts mature monocytes, neutrophils, eosinophils, and the activated T cells to the inflammatory focus [2]. 

However, the participation of this protein in the role played by the hemostatic system has not been sufficiently studied. The smooth muscle cells of vascular walls are known to secrete rhCyA under hypoxic and oxidative stress conditions; the activated platelets in the injured vascular wall are capable of secreting rhCyA [3], which in turn stimulates endotheliocyte proliferation [4], thereby facilitating the development of regenerative processes. 

The *in vitro* effect of recombinant human cyclophilin A on the growth of a fibrin clot is studied in this work. 

## EXPERIMENTAL 

Recombinant human rhCyA was produced using *Escherichia coli* BL21 cells transformed with plasmid derived from pCyPAwt/pGEX-2TK, which encodes the GST-CyPA fusion protein. The genetic construct was used with the kind permission from M. Bukrinsky (Albert Einstein College of Medicine of Yeshiva University, USA). The synthesis of GST-CyPA was induced by adding 0.2 mM isopropyl-β-D-thiogalactopyranoside until a finite concentration of 100 µM was achieved. The cells were then deposited via centrifugation, suspended in a Na-K phosphate buffer (pH 7.3), and subjected to ultrasonic destruction. The cell lysate was centrifugated; the superprecipitate was applied onto a GSTrap FF column (“GE Healthcare”). The GST-CyPA fusion protein was eluted with 50 mM Tris-HCl buffer (pH 8.0) containing 10 mM reduced glutathione. The fractions containing GST-CyPA were combined, transferred into a 10 mM Tris-HCl solution (pH 8.0) on a HiTrap Desalting column (“GE Healthcare”), and applied onto a MonoQ 5 × 50 column (“GE Healthcare”). Elution was performed using a 1 M sodium chloride gradient (0–1 M) in 10 mM Tris-HCl, pH 8.0. The fractions containing GST-CyPA were transferred via dialysis into a Na-K phosphate buffer (pH 7.3); a thrombin solution (“Sigma-Aldrich”) on the basis of a 1 U/mg fusion protein was subsequently added. The cleavage was carried out for 10 h at 4°C. The mixture of the CyPA and GST proteins was separated on a GSTrap FF column. Thrombin was removed from the CyPA solution on a HiTrap Benzamidine FF column (“GE Healthcare”). The degree of reaction completion was controlled via polyacrylamide gel electrophoresis under denaturizing conditions (SDS-PAAG). The purity of the protein used was ~98%. 

**Table:** Effect of 30-min and 3-h incubations of platelet-rich plasma with rhCyA on the parameters of the spatial dynamics of clot growth

Probe	DCG, min	IGR, min	SSGR, min	CS-30, µm	CD, arb. units
30-min incubation					
Control	3.0 ± 1.6	53.3 ± 16.7	32.8 ± 13.1	868.0 ± 180.1	13679.0 ± 2395.0
Test	14.8 ± 5.7*	14.3 ± 6.2*	16.8 ± 7.2*	341.1 ± 114.3*	9116.0 ± 2086.0*
3-h incubation					
Control	1.0 ± 0.3	59.7 ± 4.8	37.0 ± 9.0	1329.0 ± 244.7	16182.0 ± 2797.0
Test	1.4 ± 0.6	61.8 ± 6.7	33.5 ± 5.2	1258.0 ± 189.8	13971.5 ± 3294.5

Note: * – reliability of differences p <0.05 compared with the control.

The *in vitro* effect of rhCyA on the spatial dynamics of clot growth was studied using venous blood collected by gravity flow from the peripheral vein of healthy volunteers and stabilized with a 3.7% solution of sodium citrate at a 1 : 9 citrate : blood ratio. 

Blood samples were centrifugated at 1500 rpm for 7 min. The platelet-rich plasma was divided into two parts. The first probe was used as the control (C); rhCyA at a final concentration of 10 or 50 µg/ml was added into the second probe (experimental – E), followed by incubation at 37 ^о^ С for 30 min and 3 h with constant stirring to prevent platelet sedimentation. A granulocyte colony-stimulating factor (G-CSF) at a final concentration of 20 µg/ml was added as the reference substance. 

After the incubation, the platelet-rich plasma was centrifugated at 13700 rpm for 10 min. The platelet-free plasma was used in order to study the spatial dynamics of clot growth in accordance with the manual for ThromboImager-2 instrument (OOO “GemaСor”, Russia). 

The values of the delay of clot growth (the so-called lag phase) (min), the initial (min) and steady-state (min) clot growth rates, clot size after the 30 minutes of growth (µm), clot density (arbitrary units), and spontaneous thrombus formation in a chamber were recorded. 

The experimental results were statistically processed using the Mann–Whitney test. differences with *р* < 0.05 were considered to be statistically significant. 

## RESULTS 

The parameters of the spatial dynamics of clot growth upon the incubation of the platelet-rich plasma with rhCyA for 30 min and 3 h are listed in *Table* .  

It is clear from *Table * that the 30-min incubation of platelet-rich plasma with rhCyA at a final concentration of 10 µg/ml resulted in a statistically significant inhibition of the spatial dynamics of clot growth; i.e., rhCyA exhibited the properties of a substance exhibiting a hypocoagulation effect. Spontaneous thrombus formation in the volume of the measuring chamber was detected for two samples in the control group. The incubation with rhCyA prevented the development of spontaneous clot formation in the system. However, this effect lasted for a short time; the differences in the parameters of the spatial dynamics of clot growth disappeared as early as after 3 h ( *Table* ). 

No dose-dependent effect of the agent on clot growth was revealed upon incubation of the samples of platelet-rich plasma with rhCyA at the final concentration of 50 µg/ml. The parameters of the spatial dynamics of clot formation were identical to those during the incubation with rhCyA at a final concentration of 10 µg/ml. No fall in the clot growth rate was observed after 3 h of incubation. 

G-CSF had no effect on the values of the determined indices, which were comparable to those in the control group. 

## DISCUSSION 

Clot formation in the organism only occurs locally; a clot is formed in a small area of a damaged blood vessel wall. Vessel wall damage results in exposure of the tissue factor (the protein immobilized on the membrane of vessel wall cells, which are covered with an endothelium layer prior to the damage) into the blood. Diffusion of the coagulation factors plays a significant role in the spatial growth of the clot and its localization. Individual pathologies have specific effects on the activity of the components of the blood coagulation system, causing defined changes at various stages of the process. The disruptions in the mechanism of haemocoagulation manifest themselves clinically either as a haemorrhage, as thrombosis, or as a combination of these factors. 

It should be noted however that the clot formation process to a significant extent occurs in the organism under spatially heterogeneous conditions. Therefore, the role of individual coagulation reactions can differ for * in vitro* experiments under stirring and *in vivo * [5]. 

The method for recording the spatial dynamics of clot growth is based on the contact between the blood and the tissue factor in a measuring cell containing immobilized thromboplastin [6]. The activated nucleation and spatial growth of a clot is recorded on the basis of dark-field scattering microscopy. This method enables to measure such significant characteristics of the process as the clot growth rate, clot size, and spontaneous clot formation; i.e., information that is not available when using homogenous methods [7]. This allows one to perform the simultaneous and independent recording of the disruptions at all stages of the process. 

It has also been ascertained (unpublished data) that rhCyA at a dose of 10 µg does not affect the internal and external pathways of blood coagulation *in vitro* . However, an eightfold increase in the level of plasma anti-Xa activity as compared to control values was observed 10 min after the onset of incubation with rhCyA. After 30- and 60-min-long incubations with the protein, the level of anti-Xa-activity decreased to some extent; however, it was still 3 times higher than that in the control sample. Identical data were obtained in animal-model experiments. The introduction of rhCyA at a dose of 100 µg to Р815 intact mice was accompanied by a statistically significant threefold increase in the level of inhibition of the factor Xa; a 200 µg dose resulted in the inhibition of the factor Xa, corresponding to 0.6 anti-Xa U/ml. The data presented in this paper indicate that rhCyA impedes clot growth by slowing the rate at which it forms via the inhibition of the factor Xa. Clot growth does not start immediately and is a heterogeneous process; therefore, thrombus formation is less plausible, since this clot is washed off by the bloodstream in the organism [8]. 

The blood coagulation has threshold and autocatalytic properties; therefore, its spatial behavior may have much in common with the active media. In 1994, Ataullakhanov and Guriya put forth a hypothesis according to which spatial (but not homogeneous) clot formation is an autowave process [9]. A deficiency in the factors of the internal coagulation pathway or their inhibition may disrupt the autowave phase of growth. 

Autowave propagation of thrombin cannot be slowed by natural inhibitors, since they are incapable of ending an autowave because of their homogeneous spatial distribution. To provide deceleration, the autocatalytic generation of coagulation factors has to be suppressed. The hypothesis of an autowave propagation of the coagulation process involves the existence of a double autowave mechanism for the termination of the thrombin wave [10]. It is believed that the second wave of substance can be initiated in the environment; this wave emerges after thrombin formation and punctuates its synthesis. If this wave propagates at a higher speed than the first wave, it will be able to end the propagation of the first wave. 

A thrombin inhibitor capable of propagating autocatalytically thus far remains unknown. Assumptions have been made that thrombin can exist in pro- and anticoagulant forms [11]. In its procoagulant state, thrombin is highly active with respect to fibrinogen and poorly active with respect to protein C. On the contrary, thrombin in its anticoagulant form (due to the change in the conformation of thrombin conjugated with some compounds) shows a high level of activity with respect to protein C and weak activity with respect to fibrinogen [12]. Our data on the action of rhCyA on the spatial dynamics of clot growth enables to hypothesize that rhCyA might influence the mechanisms regulating the autowave process of blood coagulation. 

## CONCLUSIONS 

Recombinant human cyclophilin A exhibits a pronounced dose-independent anticoagulant effect. 

Recombinant human cyclophilin A can prevent spontaneous thrombus formation. 

The anticoagulant effect of recombinant human cyclophilin A persists for no longer than 2 h.  

## Abbreviations

rhCyA: recombinant human cyclophilin A
DCG: delay of clot growth, min
IGR: initial growth rate, min
SSGR: steady-state growth rate, min
CS-30: clot size after 30-minute growth, µm
CD: the clot density
G-CSF: granulocyte colony-stimulating factor
